# Postlumpectomy imaging: is there a role for the study of the contralateral breast?—a retrospective cohort

**DOI:** 10.1097/j.pbj.0000000000000244

**Published:** 2024-02-12

**Authors:** André M. Pires, Jéssica R. Rodrigues, Helena G. Pereira

**Affiliations:** a Radiation Oncology Department, Portuguese Institute of Oncology of Porto, Porto, Portugal; b Cancer Epidemiology Group, Portuguese Institute of Oncology of Porto, Porto, Portugal

**Keywords:** breast cancer, breast conservative therapy, residual disease, contralateral breast

## Abstract

**Background:**

Some patients with breast cancer submitted to breast-conserving surgery might benefit from a postlumpectomy imaging examination previously to radiation therapy. This aims to document the complete removal of cancer and might be accomplished using mammogram with breast and axillary ultrasonography. These modalities study not only the affected side but also the contralateral side. In fact, it is well-documented that women with breast cancer have an increased risk for contralateral breast cancer. Thus, we intended to evaluate the value of postlumpectomy imaging undertaken before adjuvant radiotherapy regarding the evaluation of the contralateral breast and axilla.

**Methods:**

In this retrospective study, medical records for patients with breast cancer submitted to breast-conserving surgery and referred to our radiotherapy unit between 2018 and 2019 were reviewed. All patients had to be submitted to bilateral mammogram with breast and axillary ultrasonography previously to radiotherapy. Patients with bilateral disease or with a history of breast cancer were excluded.

**Results:**

One thousand two hundred forty patients were analyzed. 19 (1.5%) had suspicious findings for contralateral breast disease, and 8 (0.6%) had a re-excision positive for residual malignancy. Higher age, invasive lobular carcinoma associated or not with lobular carcinoma in situ, and presence of lobular carcinoma in situ were associated with an increased risk for residual disease.

**Conclusion:**

Contralateral evaluation as part of postlumpectomy imaging revealed itself useful at detecting contralateral cancer, with some demographic and clinical features being associated with an increased risk for residual disease.

## Introduction

For women with breast cancer, the success of breast conservation therapy (BCT) relies on adequate removal of cancer, so the burden of residual malignancy is sufficiently small, and it can be sterilized by radiotherapy (RT).^1^ For assessing the adequacy of the surgical excision, we rely on several approaches, namely the histopathological evaluation of the surgical margins, intraoperative specimen radiograph, and postexcision preirradiation imaging examinations. Regarding surgical margins, the definition of a negative surgical margin has been the source of historical controversy. However, nowadays, it is recognized that the involvement of the resection margins is an important risk factor for local recurrence, so most centers rely mainly on it to confirm the complete surgical excision.^2^ On the other hand, the value of both the specimen radiograph and the postlumpectomy imaging (PLI) studies remains unclear. First, multiple studies have evaluated the accuracy of specimen radiograph in predicting margin involvement and revealed poor accuracies, with reported sensitivities between 22% and 77%.^3,4^ Second, concerning PLI, there is no solid evidence that it confers any significant clinical benefit, which explains why some centers omit their practice. Furthermore, there is no consensus on which imaging examinations should be performed, so their choice is largely justified by institutional preferences. Two of the imaging modalities available are the mammogram and ultrasound, which are performed not only on the affected side but also on the contralateral side.

It is well-documented that women with breast cancer have a greater risk of developing contralateral breast cancer (CBC),^5^ with a 5-year cumulative incidence between 1% and 1.8%, depending on tumor and treatment characteristics.^6^ This study aimed to evaluate the value of mammography with breast and axillary ultrasound undertaken before adjuvant radiotherapy in patients with conservatively managed invasive carcinoma and/or in situ carcinoma of the breast, to address its value regarding the contralateral breast and axilla, and to determine whether there is any group of patients with an increased risk for CBC where routine PLI should not be discarded.

## Materials and methods

This is a retrospective cohort study. Patients with breast cancer who received breast-conserving surgery and were referred to our External Radiotherapy Unit between January 2018 and December 2019 were reviewed. Patients were referred to our department to receive adjuvant external radiotherapy as part of the conservative therapy.

Ethical approval for this study (Ethical Committee N° CES 244/020) was provided by the Ethical Committee of IPO-Porto, Porto, Portugal, on September 3, 2020. The ethical standards displayed in the 1964 Declaration of Helsinki and its later amendments were followed.

PLI was performed at the discretion of the treating radiation oncologist. Patients with bilateral synchronous breast cancer or with a history of breast cancer were excluded. Since IPO-Porto is a referral center, about one-third of the patients were referred from other hospitals after surgery to complete treatments.

A routine bilateral digital mammogram and breast ultrasound were performed between the surgery and RT initiation in all patients analyzed. In some cases, a mammogram was substituted or followed by a breast tomosynthesis, at the discretion of the radiologist. Owing to the retrospective nature of this study, radiologists did not follow a protocol for interpreting PLI examinations. However, the imaging findings were divided into those that were indeterminate or suspicious for neoplasms (this included all lesions reported by the radiologist as “highly suspicious,” “moderately suspicious,” “suspicious,” BIRAD system classification of 4 or 5, and “indeterminate”) and those that were not suspicious for neoplasms (when none of the previous terms was reported by the radiologist). All patients had histocytologic confirmation of their suspicious and indeterminate findings by the pathology department of the institution.

Patients' clinicopathological and demographic information was collected for analysis (age, tumor phenotype on diagnostic mammogram, clinical and pathological stage, histological subtype and tumor grade, steroid hormone receptor expression, systemic treatment, date and place of surgery, and date and positive changes of PLI). This information was collected by the primary investigator of the study, using electronic clinical records. Missing data were minor, only existent for patients referenced from external institutions, and were minimized through the access of the electronic system of patients of those referring hospital. All patients also underwent a physical examination before irradiation.

Data were analyzed using IMB® SPSS® Statistics software, version 26. Continuous variables were described by their mean ± standard deviation, and categorical variables were expressed as actual numbers (n) and percentages (%). Normal distribution was checked using Kolmogorov-Smirnov and Shapiro-Wilk tests. Our data follow a normal distribution, so parametric tests were applied. Comparisons between groups were performed, using independent samples *t* tests for continuous variables. Chi-squared or Fisher exact tests were used to evaluate the association between two categorical variables. Binary logistic regression models were performed. However, owing to the low number of outcomes, only univariable models were considered. All tests of statistical significance were two-sided, and a *P* value <.05 was considered significant.

## Results

### Patients' characteristics

One thousand three hundred eighty-nine patients with breast cancer were referred to our department to receive adjuvant radiotherapy. In total, 149 patients were not submitted to PLI, so a total of 1240 patients met the selection criteria and were analyzed. Participants had a mean age of 57.2 (±11.0) years. A mass, calcifications associated with a mass and calcifications alone were present in the initial diagnostic mammogram in 71.8%, 17.0%, and 10.8%, respectively. Most women (91.8%) presented an extension of disease ≤3 cm. Five women (0.4%) presented nipple changes. Neoadjuvant therapy was performed in 14.0% of the cases.

Infiltrating ductal carcinoma and infiltrating ductal carcinoma associated with ductal carcinoma in situ were the most common subtypes identified on the initial lumpectomy (22.6% and 55.1%, respectively); 7.6% of patients had pure ductal carcinoma in situ, and 6.1% had invasive lobular carcinoma associated or not with lobular carcinoma in situ. Most tumors had differentiation of grade II or III. Most women were staged as having pT1 tumors (60.9%). In total, 86.2% of all patients were hormone receptor positive, while 14.2% and 11.1% of the invasive tumors were HER2 positive and triple negative, respectively.

The median time interval between surgery and PLI was 128.3 (±68.8) days. A summary of patient and disease characteristics is presented in Table 1.

**Table 1:** Patient characteristics (left) and characteristics according to the presence/absence of suspicious findings for contralateral residual disease (middle) and to the presence/absence of contralateral residual disease (right) on PLI

Variable	All cases (n = 1240 patients)	Suspicious findings for contralateral disease		*P*	Residual contralateral disease		*P*
		Not present (n = 1221)	Present (n = 19)		Not present (n = 1232)	Present (n = 8)	
Mean age at surgery (y)	57.2 ± 11.0	57.2 ± 10.9	56.3 ± 13.4	.728	57.1 ± 10.9	65.1 ± 11.1	**.040**
Clinical/imaging presentation				.346			.391
Microcalcifications	132 (10.8)	132 (11.0)	0 (0.0)		132 (10.9)	0 (0.0)	
Mass	876 (71.8)	863 (71.8)	13 (72.2)		871 (71.9)	5 (62.5)	
Microcalcifications + mass	207 (17.0)	202 (16.8)	5 (27.8)		204 (16.8)	3 (37.5)	
Nipple changes	5 (0.4)	5 (0.4)	0 (0.0)		5 (0.4)	0 (0.0)	
Extent at presentation				1.000			1.000
≤3 cm	1100 (91.6)	1083 (91.5)	17 (94.4)		1092 (91.5)	8 (100)	
>3.1 cm	101 (8.4)	100 (8.5)	1 (5.6)		101 (8.5)	0 (0.0)	
Neoadjuvant treatment				.095			.609
Yes	174 (14.0)	174 (14.3)	0 (0.0)		174 (14.1)	0 (0.0)	
No	1066 (86.0)	1047 (85.7)	19 (100.0)		1058 (85.9)	8 (100.0)	
Histologic subtype				**.019**			**.002**
Invasive carcinoma NST ± DCIS	964 (77.7)	954 (78.1)	10 (52.6)		961 (78)	3 (37.5)	
Pure DCIS	94 (7.6)	92 (7.5)	2 (10.5)		93 (7.5)	1 (12.5)	
ILC ± LCIS	76 (6.1)	72 (5.9)	4 (21.1)		73 (5.9)	3 (37.5)	
Other	106 (8.5)	103 (8.4)	3 (15.8)		105 (8.5)	1 (12.5)	
Presence of DCIS	836 (67.4)	822 (67.3)	14 (73.7)	.557	831 (67.5)	5 (62.5)	.720
Presence of LCIS	95 (7.7)	91 (7.5)	4 (21.1)	.051	95 (7.7)	3 (37.5)	**.018**
Invasive grade*				.608			**.022**
I	129 (11.4)	128 (11.5)	1 (5.9)		129 (11.5)	0	
II	543 (48.1)	533 (47.9)	10 (58.8)		536 (47.7)	7 (100.0)	
III	458 (40.5)	452 (40.6)	6 (35.3)		458 (40.8)	0	
In situ grade†				.765			.715
I	68 (8.2)	68 (8.2)	2 (13.3)		67 (8.2)	1 (16.7)	
II	250 (30.3)	252 (30.3)	4 (26.7)		248 (30.2)	2 (33.3)	
III	508 (61.5)	509 (61.5)	9 (60.0)		505 (61.6)	3 (50.0)	
After neoadjuvant therapy							
T status‡				—			—
ypT0	71 (40.8)	71 (40.8)	0 (0.0)		71 (40.8)	0 (0.0)	
ypTis	12 (6.9)	12 (6.9)	0 (0.0)		12 (6.9)	0 (0.0)	
Mean in situ extension (mm)	13.7 ±17.9	13.7 ±17.9	—	—	13.7 ±17.9	—	—
ypT1	71 (40.8)	71 (40.8)	0 (0.0)		71 (40.8)	0 (0.0)	
ypT2	20 (11.5)	20 (11.5)	0 (0.0)		20 (11.5)	0 (0.0)	
Multifocal	35 (20.1)	35 (20.1)	0 (0.0)	—	35 (20.1)	0 (0.0)	—
No neoadjuvant therapy							
T status‡				.429			.869
pTis	96 (9.0)	94 (9.0)	2 (10.5)		95 (9)	1 (12.5)	
Mean in situ extension (mm)	23.6 ±16.2	23.8 ±16.3	27.5 ±10.6	.748	23.7 ±16.3	35	.491
pT1	753 (70.6)	742 (70.9)	11 (57.9)		748 (70.7)	5 (62.6)	
pT2/3	217 (20.4)	211 (20.2)	6 (31.6)		215 (20.3)	2 (25.0)	
Multifocal (%)	152 (14.3)	151 (14.4)	1 (5.3)	.502	152 (14.3)	0 (0.0)	.610
Multicentric (%)	8 (0.8)	7 (0.7)	1 (5.3)	.134	7 (0.7)	1 (12.5)	.059
Receptor status							
HR-positive	1069 (86.2)	1050 (86)	19 (100)	.095	1061 (86.1)	8 (100.0)	.608
HER2 positive§	159 (14.2)	157 (14.3)	2 (13.3)	1.000	159 (14.3)	0 (0.0)	.602
Triple negative§	124 (11.1)	124 (11.3)	0 (0.0)	.396	124 (11.2)	0 (0.0)	1.000
Days from surgery to PLI				**.016**			1.000
≤100	665 (53.6)	660 (54.1)	5 (26.3)		661 (53.7)	4 (50)	
>100	575 (46.4)	561 (45.9)	14 (73.7)		571 (46.3)	4 (50)	
Time from surgery to RT start (days)‖	128 ± 68.8	127.2 ± 67.4	183.9 ± 88.1	**<.001**	127.8 ± 67.5	158.1 ± 126.1	.209

Values are mean ± standard error. Cases with missing values were removed from the analysis.

* Excludes patients without invasive component.

† Excludes patients without in situ component.

‡ Staging according to the AJCC 8th edition.

§ Excludes patients with complete pathological response to neoadjuvant chemotherapy (i.e., ypT0).

‖ Excludes patients with residual locoregional disease.

Bold values denote statistical significance at the *P* < 0.05 level.

DCIS = ductal carcinoma in situ; HR = hormone receptors; ILC = invasive lobular carcinoma; LCIS = lobular carcinoma in situ; NST = no special type; RT = radiation therapy.

### Value for detecting residual contralateral disease

Among the 1240 patients considered, 19 (1.5%) had suspicious findings. Of these, 8 (42.1%) had re-excision compatible with contralateral malignant disease. Thus, PLI identified residual CBC in 0.6% of patients overall. The characteristics of these patients are presented in Table 1.

When analyzing the different variables, only the histologic subtype (*P* = .019) and time from surgery to PLI (*P* = .016) were significantly associated with the presence of suspicious findings for CBC on PLI. On the binary logistic regression models (Table 2), both the presence of invasive lobular carcinoma associated or not with lobular carcinoma in situ (*P* = .006) and a period between surgery and PLI > 100 days (*P* = .023) were associated with an increased risk for suspicious CBC findings.

**Table 2:** Univariate analysis related to the presence of suspicious findings for residual contralateral disease on PLI

Variable	OR (95% CI)	*P*
Histologic subtype		
Invasive carcinoma NST ± DCIS	Ref.	—
Pure DCIS	2.074 (0.448–9.608)	.351
LCI ± LCIS	5.300 (1.622–17.318)	**.006**
Other	2.779 (0.753–10.258)	.125
Time from surgery to PLI		
≤100	Ref.	—
>100	3.294 (1.179–9.202)	**.023**

Bold values denote statistical significance at the *P* < 0.05 level.

DCIS = ductal carcinoma in situ; ILC = invasive lobular carcinoma; LCIS = lobular carcinoma in situ; NST = no special type.

However, when considering the predictors for contralateral disease, the number of variables significantly associated expands the following: age (*P* = .040), histologic subtype (*P* = .002), presence of lobular carcinoma in situ (*P* = .018), and the grade of the invasive component (*P* = .022). The results of univariable analysis (Table 3) show that higher age at surgery (*P* = .046), histologic subtype invasive lobular carcinoma associated or not with lobular carcinoma in situ (*P* = .002), and presence of lobular carcinoma in situ (*P* = .007) were all associated with a greater risk for CBC.

**Table 3:** Univariate analysis related to the presence of residual contralateral disease on PLI

Variable	OR (95% CI)	*P*
Age at surgery (y)	1.072 (1.001–1.149)	**.046**
Histologic subtype		
Invasive carcinoma NST ± DCIS	Ref.	—
Pure DCIS	3.444 (0.3535–33.446)	.286
ILC ± LCIS	13.164 (2.611–65.381)	**.002**
Other	3.051 (0.315–29.593)	.336
Presence of LCIS	7.320 (1.723–31.099)	**.007**

Bold values denote statistical significance at the *P* < 0.05 level.

DCIS = ductal carcinoma in situ; ILC = invasive lobular carcinoma; LCIS = lobular carcinoma in situ; NST = no special type.

## Discussion

In this study, we retrospectively reviewed our institution's experience on patients with invasive and in situ carcinoma of the breast and managed conservatively with negative margins, to evaluate the usefulness of mammography and breast and axillary US performed before RT to the contralateral site. This is the first study directly addressing the value of both mammogram and US, as all the previous papers rely on mammography alone.

Of the total study group of 1240 patients analyzed, 19 (1.5%) had suspicious findings for neoplasm. In 8 (0.6%), the findings proved to indicate neoplasm, representing a considerable predictive positive value of 42.1%. Our analysis shows that older age, the presence of invasive lobular carcinoma associated or not with lobular carcinoma in situ, and the presence of lobular carcinoma in situ were all associated with increased risk for contralateral residual disease, which is in agreement with recent studies.^7^ In fact, analyzing the characteristics of the patients with CBC, it is noteworthy that although invasive lobular carcinoma is present in only 6.1% of the population, 3 of the 8 (37.5%) patients with CBC had initially invasive lobular carcinoma, all with lobular carcinoma in situ associated. Although patients with imaging examinations done after 100 days of surgery showed more suspicious findings for CBC, this does not translate into more residual CBC.

For women who receive chemotherapy, either in a neoadjuvant or adjuvant scheme, there will frequently be a significant period, usually larger than 6–8 months, between the last image screening regarding the contralateral breast and the initiation of RT. The superior risk for CBC in these women, allied to the long interval between diagnosis and postlumpectomy imaging examinations, may explain the many cases of contralateral breast cancers diagnosed in this study.

We consider these are not negligible findings. Contralateral PLI leads to an early diagnosis of CBC in an important portion of the patients. Previous studies reported that the early development of CBC is associated with worse survival.^8,9^ As PLI allows for an early diagnosis of CBC, it may lead to improved survival, which led us to believe routine use of PLI is beneficial in the setting of detecting contralateral disease.

Because we practice in an era limited by cost containment and sometimes reduced resources, we also recognize it may be impractical for every hospital to perform it in each patient submitted to breast conservative surgery with negative margins. Older patients, patients with invasive lobular carcinoma, and patients with a lobular carcinoma in situ component are more likely to present a CBC and, therefore, should not discard PLI.

This study has a few limitations. First, as a retrospective study, the results are subject to selection bias. As PLIs were not routinely performed in all patients, but rather at the description of the radiation oncologist, some patients with initial stages or very good prognosis features may have not been submitted to PLI. Second, although this study has a large population, the distribution between groups is considerably different (i.e., 1221 vs. 19 on suspicious findings disease and 1232 vs. 8 on residual disease), which restrains the statistical analysis.

## Conclusions

Postlumpectomy mammography and breast ultrasonography detected residual contralateral breast cancer in a substantial number of cases (0.6%). We believe that the study of the contralateral breast as part of the PLI study may be beneficial for every woman, particularly in some with special demographic and clinical features as we stated in this document. The major question that remains is whether PLI changes the outcomes in overall and relapse-free survival, which can only be answered with further prospective multi-institutional studies.
